# Supplementary material for: The Effect of Vice–Virtue Bundles on Consumers’ Purchase Intentions for Vice Packaged Foods: Evidence from Randomized Experiments
Source: Foods. 2023 Aug 31;12(17):3270. doi: 10.3390/foods12173270 (PMC10486616; doi:10.3390/foods12173270)
Supplement: Supplementary file 1 [file foods-12-03270-s001.zip › foods-2548823-supplementary.pdf]

Supplementary Material

1. Demographics of the all experiments

Table S1. Demographics of all experiments

Experiment		Experiment 1			Experiment 2			Experiment 3			Follow-up experiment		
condition		total	control	experimental	total	control	experimental	total	control	experimental	total	control	experimental
number		172	88	84	169	83	86	249	136	113	153	76	77
age	M_{age}(SD)	24.70(4.74)	24.57(5.22)	24.85(4.19)	25.32(5.06)	25.18(5.71)	25.45(4.36)	24.59(4.43)	24.14(4.02)	25.15(4.83)	30.90(4.67)	30.99(4.66)	30.82(4.70)
gender	% male	55.2%	52.3%	58.3%	57.4%	59.0%	55.8%	35.3%	35.3%	35.4%	58.8%	57.9%	59.7%
	% famale	44.8%	47.7%	41.7%	42.6%	41.0%	44.2%	64.7%	64.7%	64.6%	41.2%	42.1%	40.3%
educational background	below bachelor	21	12	9	18	7	11	19	10	9	10	5	5
	bachelor	132	68	64	119	59	60	191	105	86	72	39	33
	master	17	8	9	21	14	7	24	10	14	66	28	38
	PhD	2	0	2	11	3	8	15	9	6	5	4	1
monthly consumption (excluding home loans and rent)	Less than 500 yuan	2	2	0	2	1	1	1	1	0	0	0	0
	500-1500 yuan	77	40	37	47	22	25	102	53	49	22	12	10
	1500-3000 yuan	62	30	32	80	38	42	89	50	39	35	16	19
	3000-5000 yuan	21	12	9	19	9	10	32	16	16	65	35	30
	more than 5000 yuan	10	4	6	21	13	8	25	14	11	31	13	18

2. The participants and procedures of the experiments

Experiment 1

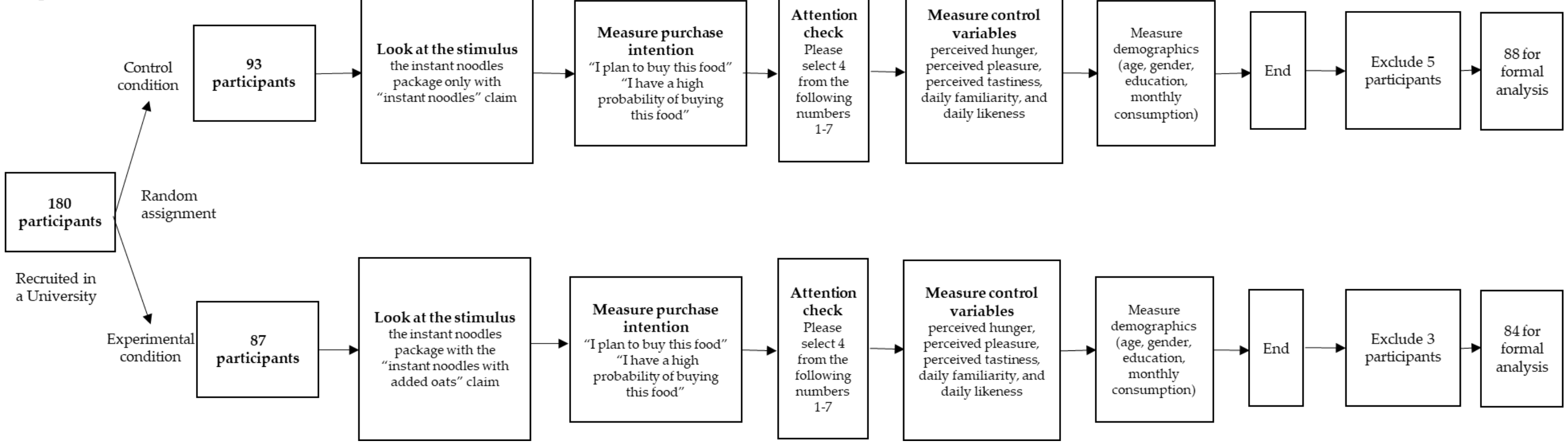

Figure S1. The participants and procedures of Experiment 1

Experiment 2

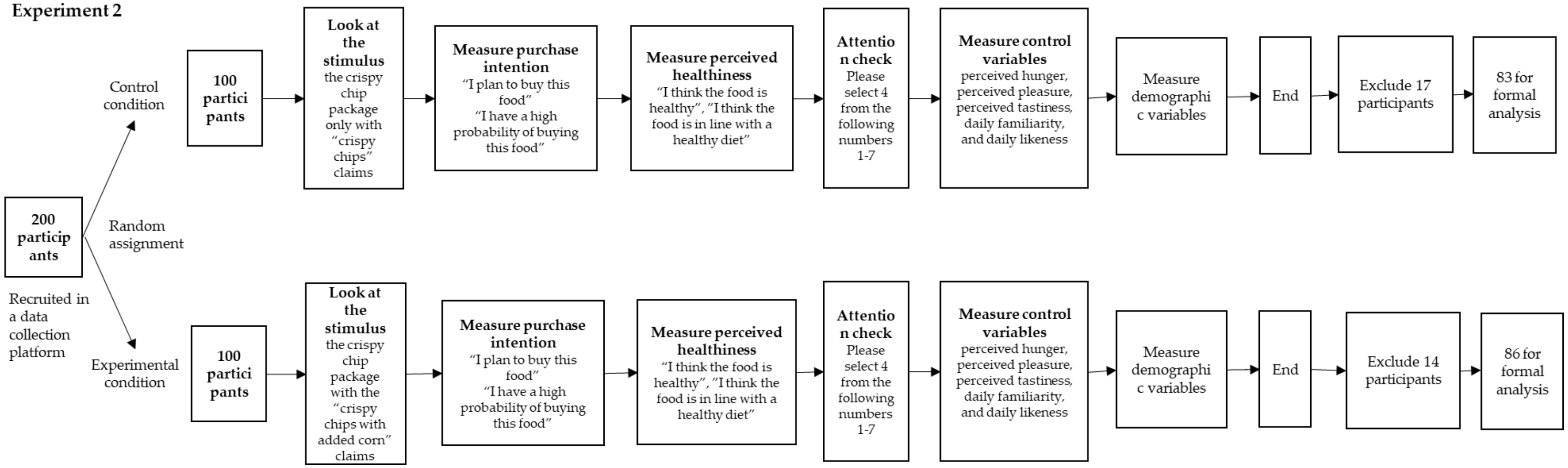

Figure S2. The participants and procedures of Experiment

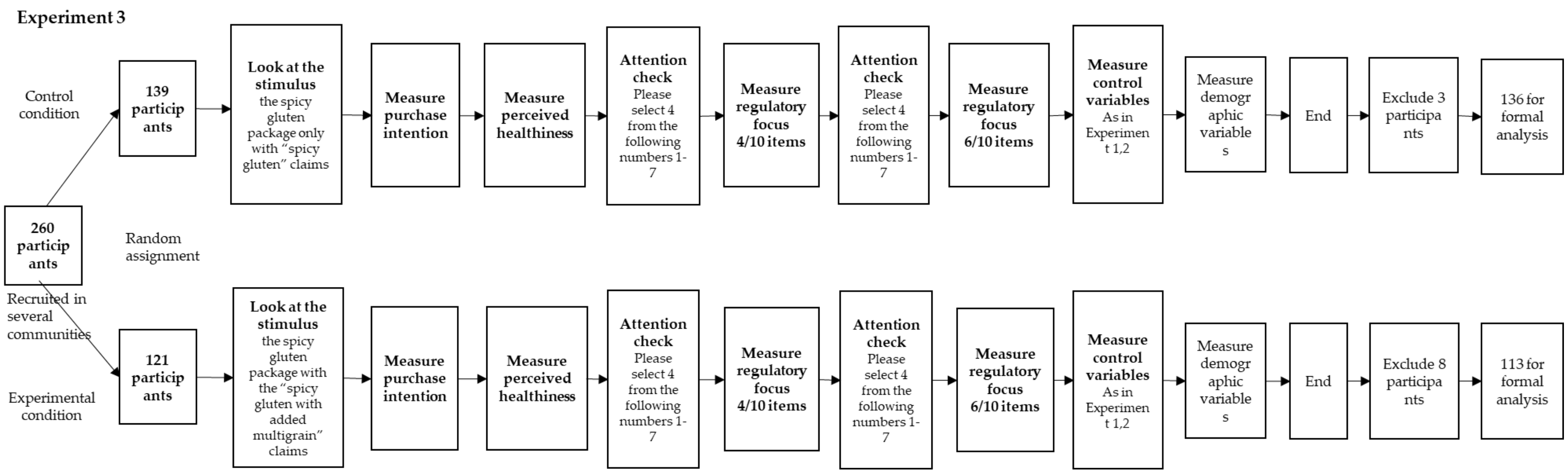

Figure S3. The participants and procedures of Experiment 3

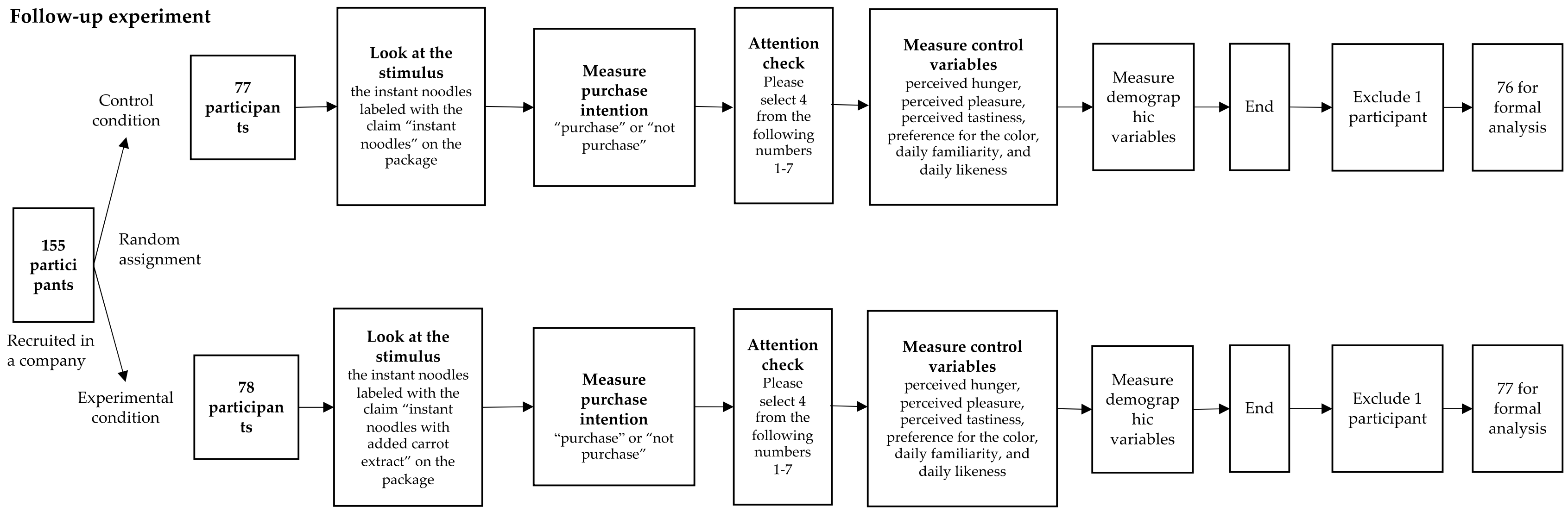

Figure S4. The participants and procedures of follow-up experiment

Note. Some participants were excluded from the formal analysis because they failed an attention check.

3. Follow-up experiment

This experiment aimed to replicate the results of Experiment 1 by using real packaged foods as stimuli and examining among company staff.

3.1. Method

3.1.1. Participants

Our researchers posted an enrollment notice to recruit participants on August 9, 2023 and closed recruitment by August 11, 2023 in a company. A total of 155 staff from this company completed the experiment between August 11 and August 14, 2023 at the company. Moreover, all participants at the time of the examination had no psychiatric diseases or any deadly diseases and they possessed normal vision. We excluded two participants for failing an attention check (e.g., "Choose the fifth option for this question"), leaving 153 participants (M_{age} = 30.90, SD = 4.67; 41.20% males). This experiment utilized a single factor (vice packaged food: virtue labelled vs. unlabelled) between-subjects design, where participants were randomly assigned to the experimental (i.e., virtue labelled; n = 77) or control (i.e., unlabelled; n = 76) condition.

3.1.2. Materials and procedures

The research assistant completed the identical randomized assignments as in Experiment 1. After starting the experiment, we informed participants that they would be paid ten RMB for completing the experiment and would have the option to use three RMB to purchase one instant noodles they saw. Subsequently, participants were presented with a real packaged food. Participants in the experimental condition saw the instant noodles labeled with the claim "instant noodles with added carrot extract" on the package, while participants in the control condition saw the instant noodles labeled with the claim "instant noodles" on the package. The then were asked to make a choice of purchasing or not purchasing it. Subsequently, they then were asked to make a choice of purchasing or not purchasing it. Notably, we conducted a pretest (n = 42) to ascertain if participants' perceptions were aligned with our expectations regarding the chosen stimuli. In this pretest, we provided participants with explanations of virtue and vice in the context and requested them to evaluate their perception of the food they were looking at (1 = I perceive that the food is vice, 7 = I perceive that the food is virtue). The analysis of t-test confirmed that participants perceived instant noodles as a vice (M = 2.95, SD = 1.17, vs. 4 [scale midpoint]; $t(41) = 4.16$, $p < 0.001$) and perceived carrot as a virtue (M = 5.45, SD = 1.25, vs. 4 [scale midpoint]; $t(41) = 7.51$, $p < 0.001$). Next, we measured several control variables on a 7-point scale (1 = not at all, 7 = extremely), including perceived hunger, perceived happiness, perceived tastiness of the presented instant noodles, how much you like the package color of the instant noodles, perceived familiarity with instant noodles in daily life, and how much they like instant noodles in daily life [9]. Finally, participants answered demographic questions.

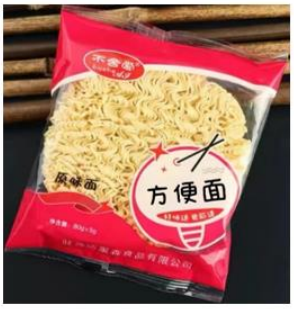

Instant noodles

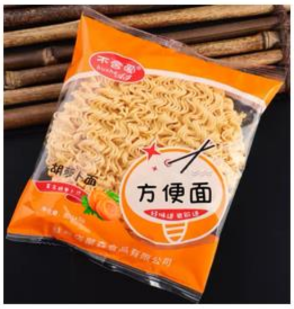

Instant noodles with added carrot extract

Note. In the left picture, the text on the left reads "original flavor instant noodles" and "net content: 80g±5g", the text at the bottom reads "Zhumadian Jusen Food Company Limited", and the text on the right reads "instant noodles". In the right picture, the text on the left reads "carrot instant noodles", "net

content: 80g±5g”, and “rich in carrot extract”, the text at the bottom reads “Zhumadian Jusen Food Company Limited”, and the text on the right reads “instant noodles”.

Figure S5. The packaged food used in the follow-up experiment.

3.2. Results

The chi-square test showed a significant difference in purchasing behavior (purchase vs. not purchase) between the control (i.e., exposed to the instant noodles without labelled carrot) and experimental (i.e., exposed to the instant noodles with labelled carrot) conditions (χ^2 = 9.28, p = 0.002). Specifically, 48.7% of participants in the control condition opted to purchase the instant noodles, while 72.7% of participants in the experimental condition opted to purchase. Thus, H1 was repeatedly supported.
